# Supplementary material for: Unlike Chloroquine, Mefloquine Inhibits SARS-CoV-2 Infection in Physiologically Relevant Cells
Source: Viruses. 2022 Feb 11;14(2):374. doi: 10.3390/v14020374 (PMC8874959; doi:10.3390/v14020374)
Supplement: Supplementary file 1 [file viruses-14-00374-s001.zip › viruses-1509221-supplementary.pdf]

Supplementary Information

Unlike Chloroquine, mefloquine inhibits SARS-CoV-2 infection in physiologically relevant cells

Short title: SARS-CoV-2 is susceptible to mefloquine in Calu-3 cells.

Carolina Q. Sacramento[1,7#*], Natalia Fintelman-Rodrigues[1,7#], Suelen S. G. Dias[1], Jairo R. Temerozo[2,3], Aline de Paula D. Da Silva[1,7], Carine S. da Silva[1,7], Camilla Blanco[1,7], André C. Ferreira[1,4,7], Mayara Mattos[1,7], Vinicius C. Soares[1,5], Filipe Pereira-Dutra[1], Milene D. Miranda[6], Debora F. Barreto-Vieira[8], Marcos Alexandre N. da Silva[8], Suzana S. Santos[9], Mateo Torres[9], Otávio Augusto Chaves[1,7], Rajith K R Rajoli[10], Alberto Paccanaro[9,11], Andrew Owen[10], Dumith Chequer Bou-Habib[2,3], Patrícia T. Bozza[1], Thiago Moreno L. Souza[1,7*]

# - These authors contributed equally to this work

1 – Laboratório de Imunofarmacologia, Oswaldo Cruz Institute, Fundação Oswaldo Cruz (Fiocruz), Rio de Janeiro, RJ, Brazil.

2 – National Institute for Science and Technology on Neuroimmunomodulation (INCT/NIM), Oswaldo Cruz Institute, Fiocruz, Rio de Janeiro, RJ, Brazil.

3 – Laboratory on Thymus Research, Oswaldo Cruz Institute, Fiocruz, Rio de Janeiro, RJ, Brazil.

4 - Universidade Iguaçu, Nova Iguaçu, RJ, Brazil.

5 – Program of Immunology and Inflammation, Federal University of Rio de Janeiro (UFRJ), Rio de Janeiro, RJ, Brazil.

6 – Laboratório de Vírus Respiratório e do Sarampo, Oswaldo Cruz Institute, Fiocruz, Rio de Janeiro, RJ, Brazil.

7 - National Institute for Science and Technology on Innovation in Diseases of Neglected Populations (INCT/IDPN), Center for Technological Development in Health (CDTS), Fiocruz, Rio de Janeiro, RJ, Brazil.

8 - Laboratório de Morfologia e Morfogênese Viral, Oswaldo Cruz Institute, Fiocruz, Rio de Janeiro, RJ, Brazil.

9 - School of Applied Mathematics, Fundação Getulio Vargas, Rio de Janeiro, RJ, Brazil

10 - Centre of Excellence in Long acting Therapeutics (CELT), Department of Pharmacology and Therapeutics, University of Liverpool, Liverpool, UK

11- Department of Computer Science, Royal Holloway, University of London, Egham, United Kingdom

Table S1. Mefloquine PBPK input parameters.

Parameter	Mefloquine
Molecular weight	378.3 [11]
Protein binding	98% [12]
Log P	3.87 [12]
pKa (diprotic base)	8.7 [12]
Blood-to-plasma ratio	1.5 [12]
Effective permeability	6.48×10^{-4} cm/s [12]
Intrinsic clearance	10.47 L/h [12]
Volume of distribution	10.2 L/kg*
Half-life	2-4 weeks [11]

*Computed using Rodgers, Leahy and Rowland equation [1].

Table S2. Mefloquine average EC_{90} values from various literature sources and experimental settings.

Cell type	EC_{90} (ng/ml)	Reference
Vero-E6	2513.8	[10]
Vero-E6	2455.2	[9]
Vero-E6	3064.3	[8]
Vero-E6	1210.6	experimental
Average (Vero-E6)	2311.0	
Calu-3	2005.1	experimental

Table S3. Physical-chemical properties of chloroquine, hydroxychloroquine, and mefloquine.

Properties[a]	Chloroquine	Hydroxychloroquine	Mefloquine
	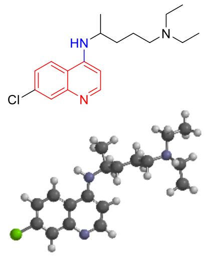	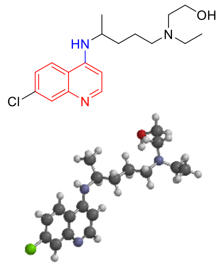	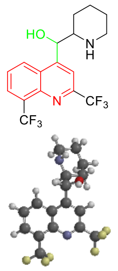
**Chemical formula**	$C_{18}H_{26}ClN_3$	$C_{18}H_{26}ClN_3O$	$C_{17}H_{16}F_6N_2O$
**Molecular weight (g/mol)**	319.9	335.9	378.31
**LogP**	4.63	4.12	3.90
**LogP (pH = 7.4)**	-0.07	-0.92	1.90
**LogP (pH = 4.0)**	-0.56	-1.42	1.90

**pKa_1**	4.0	< 4.0	< 3.0
**pKa_2**	8.4	8.3	9.46
**pKa_3**	10.2	9.7	13.79
**Area (Å^2)**	376.99	384.16	346.71
**Volume (Å^3)**	350.57	358.41	326.72
**Polar surface area (Å^2)**	18.626	35.995	36.828
**Ovality**	1.57	1.57	1.51
**Polarizability**	67.93	68.56	66.06
**Hydrogen donor count**	1	2	2
**Energy HOMO (eV at pH 7.4)**	-6.4 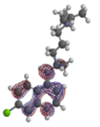	-6.4 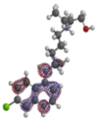	-6.9 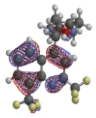
**Energy LUMO (eV at pH 7.4)**	-2.0 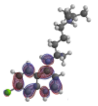	-2.0 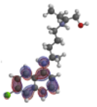	-2.2 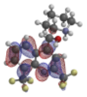
**Energy HOMO-1 (eV at pH 7.4)**	-7.2 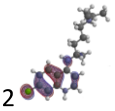	-7.2 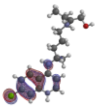	-7.4 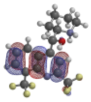
**Energy LUMO+1 (eV at pH 7.4)**	-1.0 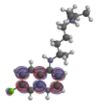	-1.0 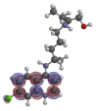	-1.1 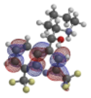
**Hydrogen acceptor count**	3	4	3
**Rotatable bond count**	8	9	4
**Melting point (°C)**	87-89.5	89-91	250-254
**Water solubility (mg/mL)**	0.0175	0.0261	0.0380
**Number of rings**	2	2	3
**Physiological charge**	+2	+2	+1
**Lipinski's rule of five**	Yes	Yes	Yes
**Chemical taxonomy**	This compound belongs to the class of organic compounds known as 4-aminoquinolines. These are organic compounds containing an amino group attached to the 4-position of a quinoline ring system.	This compound belongs to the class of organic compounds known as 4-aminoquinolines. These are organic compounds containing an amino group attached to the 4-position of a quinoline ring system.	This compound belongs to the class of organic compounds known as 4-quinolinemethanol. These are organoheterocyclic compounds containing a quinoline moiety substituted at the 4-position with a methanol.
**Plasma protein binding**	46-74%	50%	> 98%

[a]Data obtained from DrugBank; PubChem; Warhurst, et al JAC 2003; Schroeder and Gerber Toxicol. Rep. 2014; Spartan' 18 software (DFT approximation).

Table S4. KEGG pathways significantly up-regulated by mefloquine's treatment. We ran GSEA enrichment analysis with mefloquine gene expression signature from CMAP. Out of 162 KEGG pathways, ten were found to be up-regulated (FDR < 0.05). For each of these, we report the number of genes involved in the pathway that are on CMAP dataset (column "size"). In addition, we report the GSEA enrichment score (ES), the normalized enrichment score (NES), the nominal p-value testing whether ES is larger than zero (NOM p-val), and the adjusted p-value by the false detection rate (FDR q-val).

NAME	SIZE	ES	NES	NOM p-val	FDR q-val
KEGG_PROTEIN_EXPORT	17	0.716	2.491	0.000	0.000
KEGG_VIBRIO_CHOLERAE_INFECTION	48	0.479	2.239	0.000	0.005

KEGG_BIOSYNTHESIS_OF_UNSATURATED_FATTY_ACIDS	19	0.604	2.191	0.005	0.004
KEGG_LYSOSOME	109	0.405	2.142	0.000	0.004
KEGG_STEROID_BIOSYNTHESIS	16	0.595	2.060	0.000	0.005
KEGG_EPITHELIAL_CELL_SIGNALING_IN_HELICOBACTER_PYLORI_INFECTION	60	0.404	2.001	0.000	0.008
KEGG_SPLICEOSOME	106	0.346	1.915	0.000	0.013
KEGG_PROPANOATE_METABOLISM	27	0.471	1.852	0.010	0.019
KEGG_RIBOSOME	84	0.341	1.807	0.000	0.023
KEGG_INOSITOL_PHOSPHATE_METABOLISM	49	0.362	1.688	0.007	0.044

Table S5. KEGG endocytosis-related pathways down-regulated by SARS-CoV-2 infection. We ran GSEA enrichment analysis with gene expression signature of SARS-CoV-2 infection in Calu-3 cell lines. Out of six endocytosis-related pathways up-regulated by mefloquine, two are significantly down-regulated by SARS-CoV-2 infection. For each pathway, we report the number of corresponding genes available on the expression dataset (size), the GSEA enrichment score (ES), the normalized enrichment score (NES), the nominal p-value testing if ES is smaller than zero (NOM p-val), and the adjusted p-value by the false detection rate (FDR q-val).

NAME	SIZE	ES	NES	NOM p-val	FDR q-val
KEGG_PROTEIN_EXPORT	24	-0.638	-1.951	0.002	0.002
KEGG_STEROID_BIOSYNTHESIS	15	-0.623	-1.674	0.010	0.026

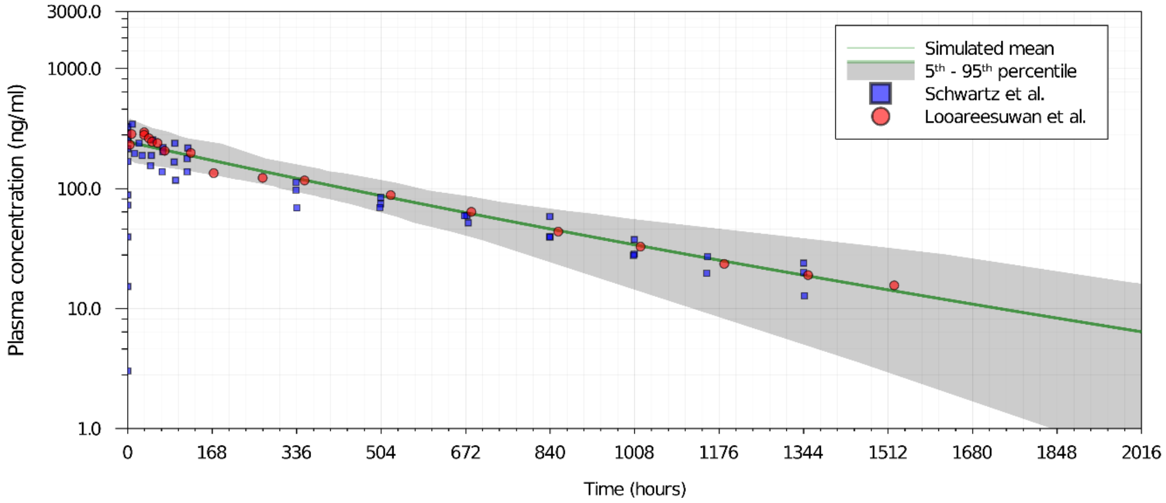

Figure S1. Mefloquine PBPK model validation against clinical data [2,3] for a single 250 mg dose.

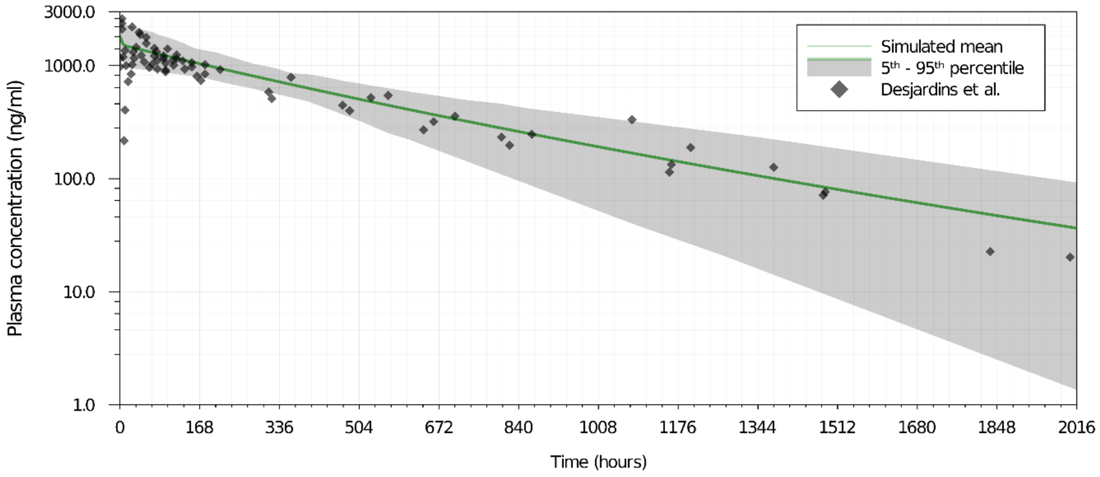

Figure S2. Mefloquine PBPK model validation against clinical data [4] for a single 1500 mg dose.

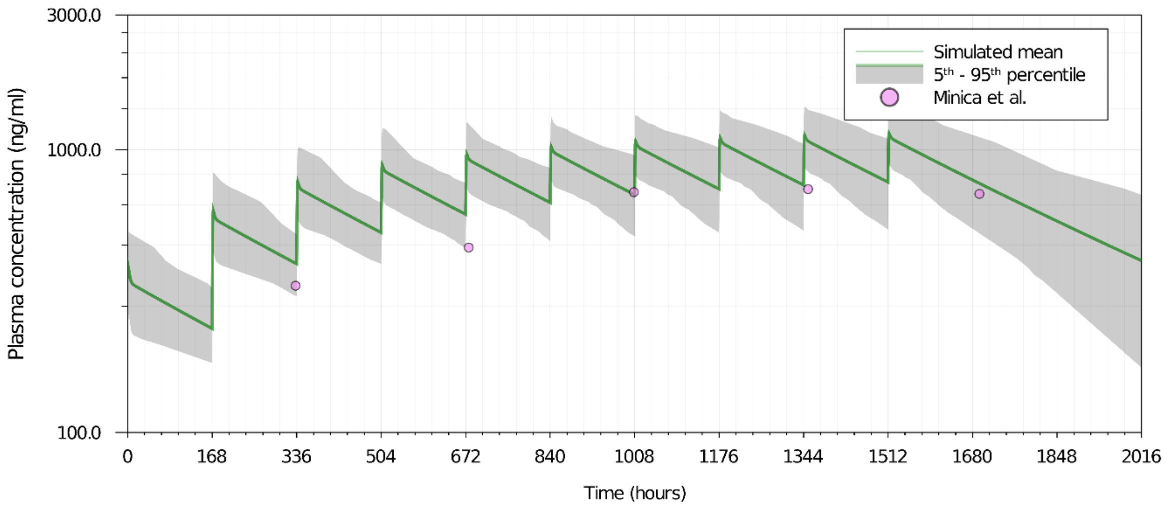

Figure S3. Mefloquine PBPK model validation against clinical data [5] for 250 mg doses administered once every week for 10 weeks.

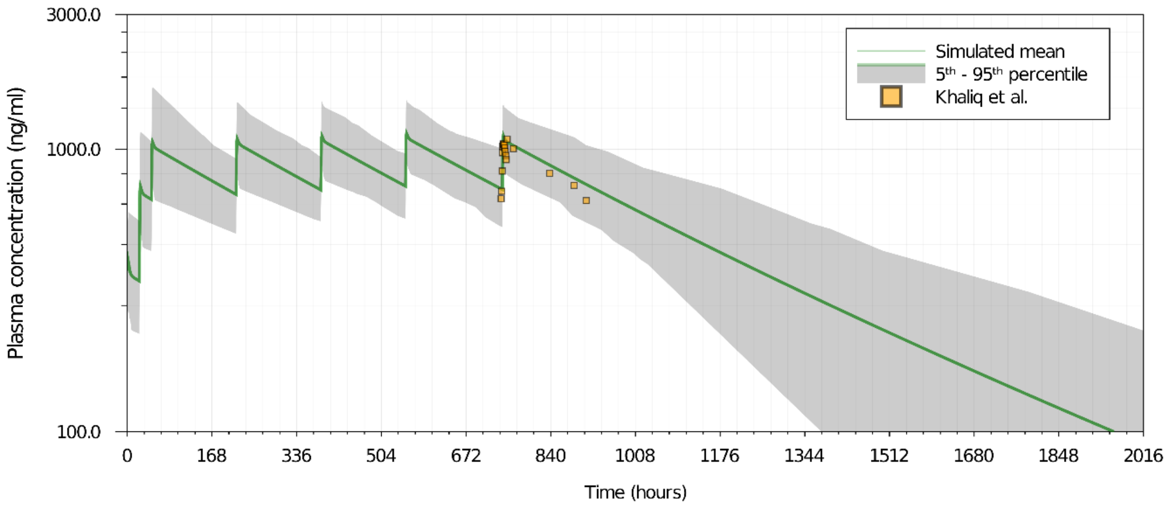

Figure S4. Mefloquine PBPK model validation against clinical data [6] for 250 mg doses administered once on day 0, 1, 2 followed by once weekly for 5 weeks.

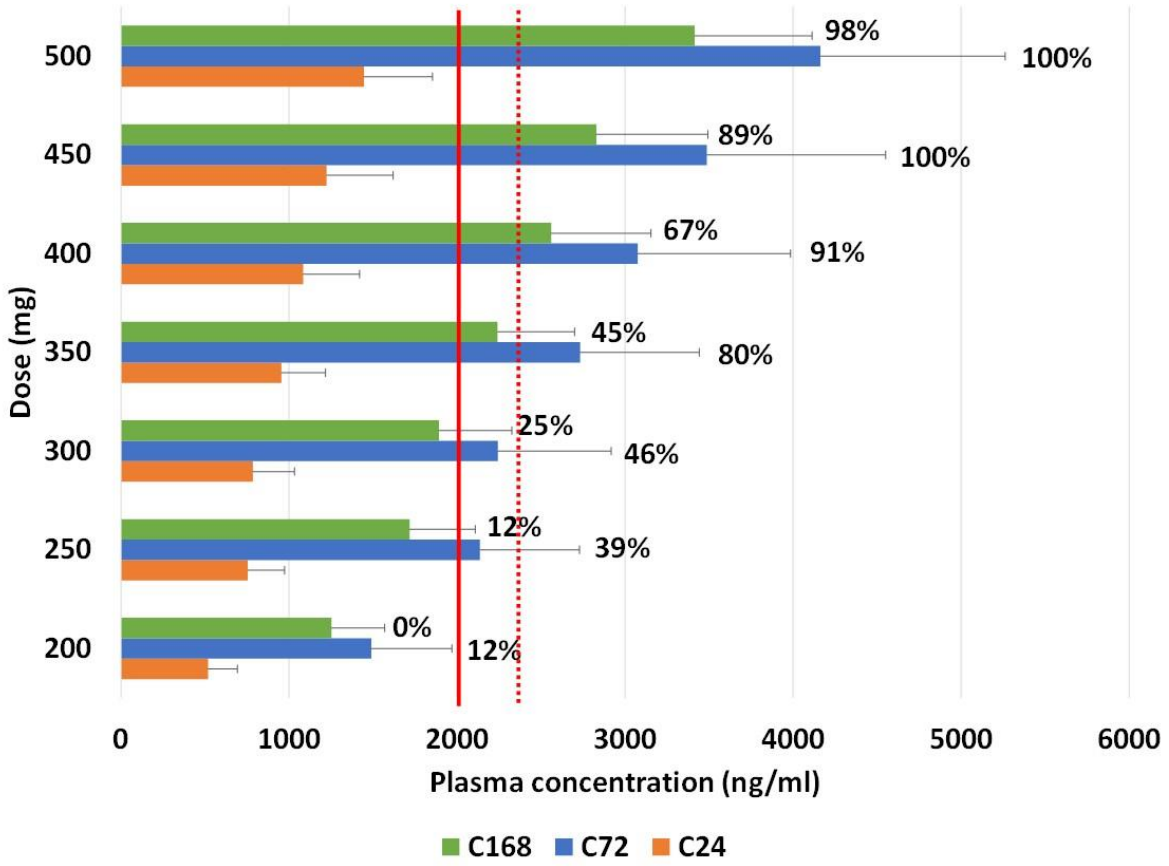

Figure S5. Median plasma trough concentrations of mefloquine on day 1 (C24), 3 (C72) and 7 (C168) for various doses administered TID for 3 days. The solid and the dotted red lines represents the EC_{90} values of mefloquine in Calu-3 and Vero-E6 cells for SARS-CoV-2, respectively. The numbers adjacent to each of the bars indicate the percentage of simulated population having plasma concentrations over the target EC_{90} value in Vero-E6 cells.

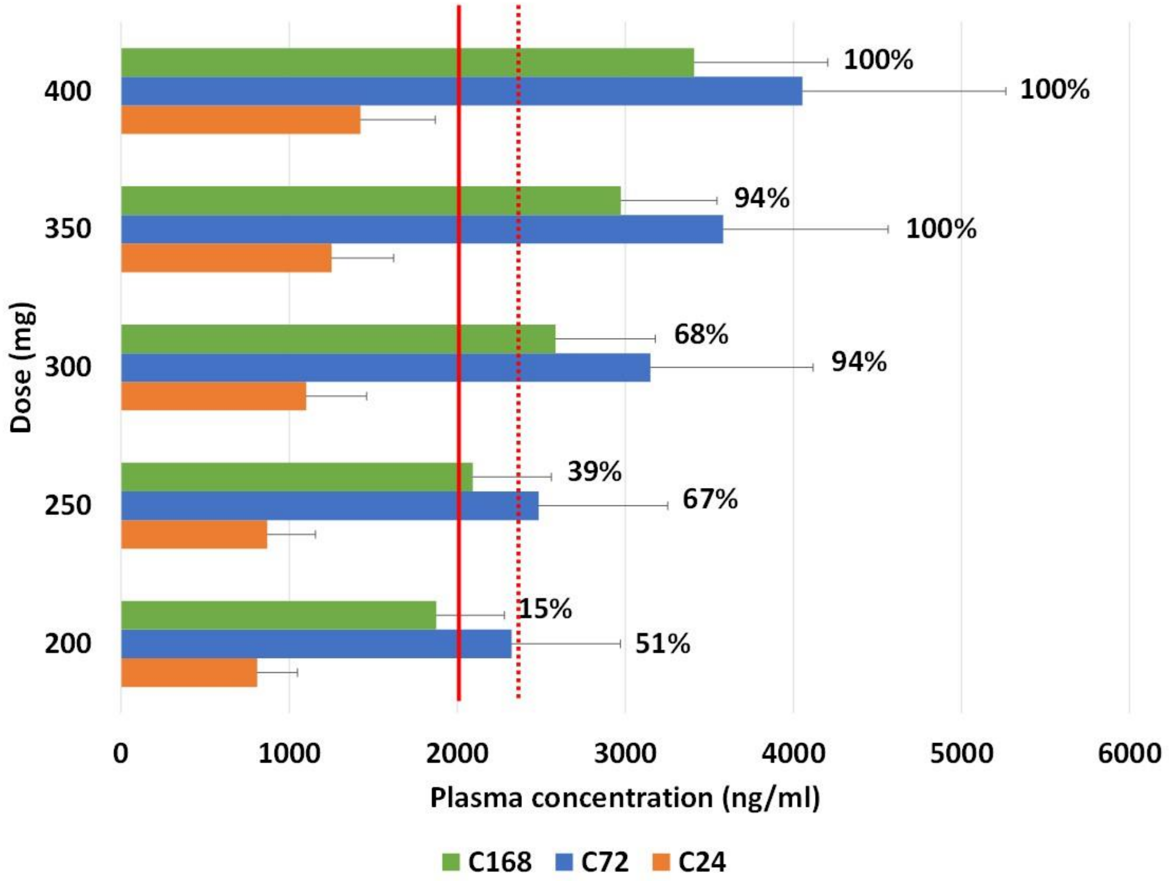

Figure S6. Median plasma trough concentrations of mefloquine on day 1 (C24), 3 (C72) and 7 (C168) for various doses administered QID for 3 days. The solid and the dotted red lines represents the EC_{90} values of mefloquine in Calu-3 and Vero-E6 cells for SARS-CoV-2, respectively. The numbers adjacent to each of the bars indicate the percentage of simulated population having plasma concentrations over the target EC_{90} value in Vero-E6 cells.

References:

1. Rodgers, T.; Leahy, D.; Rowland, M. Physiologically Based Pharmacokinetic Modeling 1: Predicting the Tissue Distribution of Moderate-to-Strong Bases. *J Pharm Sci* **2005**, *94*, 1259–1276, doi:10.1002/jps.20322.
2. Schwartz, D.E.; Eckert, G.; Hartmann, D.; Weber, B.; Richard-Lenoble, D.; Ekue, J.M.; Gentilini, M. Single Dose Kinetics of Mefloquine in Man. Plasma Levels of the Unchanged Drug and of One of Its Metabolites. *Chemotherapy* **1982**, *28*, 70–84, doi:10.1159/000238062.
3. Looareesuwan, S.; White, N.J.; Warrell, D.A.; Forgo, I.; Dubach, U.G.; Ranalder, U.B.; Schwartz, D.E. Studies of Mefloquine Bioavailability and Kinetics Using a Stable Isotope Technique: A Comparison of Thai Patients with Falciparum Malaria and Healthy Caucasian Volunteers. *Br J Clin Pharmacol* **1987**, *24*, 37–42, doi:10.1111/j.1365-2125.1987.tb03133.x.
4. Desjardins, R.E.; Pamplin, C.L.; von Bredow, J.; Barry, K.G.; Canfield, C.J. Kinetics of a New Antimalarial, Mefloquine. *Clin Pharmacol Ther* **1979**, *26*, 372–379, doi:10.1002/cpt1979263372.
5. Mimica, I.; Fry, W.; Eckert, G.; Schwartz, D.E. Multiple-Dose Kinetic Study of Mefloquine in Healthy Male Volunteers. *Chemotherapy* **1983**, *29*, 184–187, doi:10.1159/000238195.
6. Khaliq, Y.; Gallicano, K.; Tisdale, C.; Carignan, G.; Cooper, C.; McCarthy, A. Pharmacokinetic Interaction between Mefloquine and Ritonavir in Healthy Volunteers. *Br J Clin Pharmacol* **2001**, *51*, 591–600, doi:10.1046/j.1365-2125.2001.01393.x.
